# The American Psychosomatic Society – integrating mind, brain, body and social context in medicine since 1942

**DOI:** 10.1186/s13030-017-0096-6

**Published:** 2017-04-08

**Authors:** Christoph Herrmann-Lingen

**Affiliations:** 1 American Psychosomatic Society, 6728 Old McLean Village Drive, McLean, VA 22101-3906 USA; 2 Department of Psychosomatic Medicine and Psychotherapy, University of Göttingen Medical Centre, Von-Siebold-Str. 5, Göttingen, D-37075 Germany

**Keywords:** American Psychosomatic Society, Psychosomatic medicine, History

## Abstract

**Background:**

The American Psychosomatic Society is one of the oldest and probably the most influential scientific society in psychosomatic/biopsychosocial research worldwide. The current article delineates the historical development and current strategic orientation of the society.

**Method:**

Review of published literature, archived materials and current documents of the society.

**Results:**

The American Psychosomatic Society (APS) was founded in 1942, originally named the “American Society for Research in Psychosomatic Problems “. It originated from the editorial board of the Journal *Psychosomatic Medicine,* which had already been founded in 1939 and has become one of the major journals in the field. As an organization, APS has developed into a premier international scientific society, providing an interdisciplinary home for researchers from medicine, psychology and related areas, gathering under the mission “to advance and integrate the scientific study of biological, psychological, behavioral and social factors in health and disease” and dedicated to the goals of Scientific Excellence, Clinical Relevance, and Vibrant and Diverse Membership. Besides editing Psychosomatic Medicine, the APS organizes Annual Meetings and specialized events, issues several scientific awards and scholarships and is engaged in collaborative efforts to improve the research and funding landscape for biobehavioral research in the US and translate psychosomatic research findings into medical education and clinical practice.

**Conclusion:**

In its 75^th^ anniversary year, the American Psychosomatic Society has developed into the scientific landscape of the 21^st^ century, and its current updated strategy addresses contemporary demands in advancing science and improving holistic patient care.

## The beginnings

The origin of Psychosomatic Medicine in the United States of America is often attributed to Helen Flanders Dunbar of New York and her publication in 1935 of “Emotions and Bodily Changes, A Survey of Literature on Psychosomatic Interrelationships 1910-1933” [1]. On almost 600 pages, Dunbar addressed theoretical and methodological foundations of psychosomatic research before reviewing the literature on psychosomatic aspects of organs, organ systems, and their disorders and concluding with some therapeutic considerations. More than 100 pages are devoted to an exhaustive bibliography covering an enormous 2251 references available at that time.

In the Introduction, Dunbar starts by stating that “Scientific study of emotion and of the bodily changes that accompany diverse emotional experience marks a new era in medicine. We know now that many physiological processes which are of profound significance for health, not only of the individual but also of the group, can be controlled by way of the emotions. In this knowledge we have the key to many problems in the prevention and treatment of illness, yet we have scarcely begun to use what we know… Furthermore, we have reached a point where progress in the specialties themselves is being blocked by a lack of understanding of the relationships between them” ([1], p. xi).

When turning to organ systems, It appeared logical for the author to put the nervous system first, arguing that “It is customary to assume that the seat of the psyche is in the brain; … In glancing through the succeeding chapters, however, the reader will find it to have been said with equal emphasis that the seat of the psyche is in the heart, or in the *ϕρήν*, or in the ‘guts,’ etc. … In other words, study of any organ-system reveals the psyche there, and we find ourselves forced to give up the idea of a ‘localization’ of the psyche in any particular part of the body” ([1], p. 117).

Few years later, in 1939, Dunbar was the driving force behind the foundation of the Journal “*Psychosomatic Medicine*”. She became the first managing editor and a member of the editorial board representing psychiatry, together with the Chicago-based psychoanalyst Franz Alexander, Howard Liddell, representing comparative physiology, the internist Dana Atchley of New York and the pediatrician Grover Powers from Yale. Stanley Cobb represented neurology, Hallowell Davis physiology, and Clark Hall psychology. Theodore P. Wolfe was appointed as review editor without specialty assignment [2]. In the introductory statement the authors of the new Journal outlined the area of Psychosomatic Medicine as follows:

“Psychosomatic Medicine is not equivalent with what is understood by the term psychiatry. Psychiatry is concerned with the study and therapy of the disturbances of the mind whether these disturbances are the results of emotional experiences, or of anatomical changes (degenerative, inflammatory processes, or neoplasms) of the central nervous system. The principal interest of psychiatry is the diseased mind. Psychosomatic Medicine covers a different and broader field. Its object is to study in their interrelation the psychological and physiological aspects of all normal and abnormal bodily functions and thus to integrate somatic therapy and psychotherapy.

Psychosomatic Medicine is not restricted to any specific field of pathology. Medical specialties such as internal medicine, pediatrics, dermatology, ophthalmology, etc., may be so restricted. Psychosomatic Medicine, however, is not a medical specialty of this kind; it designates a method of approach to the problems of etiology and therapy rather than a delimitation of the area" ([3]; p. 3).

In its first years, the journal Psychosomatic Medicine was jointly controlled by the Editorial Board and the funders of the Journal, namely the Josiah Macy, Jr., Foundation and the US National Research Council. The first issue of the Journal went out to almost 100 subscribers, just 8 months before the beginning of World War II. Under increased economic pressure produced by the U.S. involvement in the war, both personal and material resources for the production of the journal became more and more limited and dissatisfaction with the sharing of responsibilities between the Editorial Board and the journal funders increased, leading Dunbar to proposing the foundation of an “American Society for Research in Psychosomatic Problems” as an organization to lend institutional stability to the journal [2].

There was some controversial discussion about founding a new society and some people “suggested that this should be postponed in view of the present emergency”. However, a large majority stated that “because of the present emergency and the demand for further research in psychosomatic problems no time should be lost.” [4]. In a 1959 article in JAMA, the medical historian Gregory Zilboorg [5] retrospectively argued that “from the standpoint of historical perspective, the development of psychosomatic medicine was not necessarily a result of World War II” but that “born out of historical necessity, psychosomatic medicine remains overburdened with new problems which medicine seems to be unable to solve and which psychiatry is as yet able merely to give names.”

The new society held its inaugural meeting in New York City on December 18, 1942, with a main thematic focus on fatigue [4]. Instead of an expected 50 participants (due to war-related government restrictions on travel and the short notice given of the meeting), almost 300 persons attended the one-day meeting. At the following meeting in May, 1943, Tracy Putnam, a Boston Neurologist, became the first President of the society and several other officers and Council members were elected. Since then, presidents changed annually, but although the initiator of the society had been a woman, she never became president and it lasted until 1972 that Margaret Singer became the first female – and also the first Ph.D. – President [6].

In the first years, both the Journal and the Society – despite their explicitly interdisciplinary self-concept - were mainly influenced by psychoanalytically trained psychiatrists. After the end of the war, the interest in psychosomatic medicine increased substantially, as did subscriptions to the Journal, which had approximately 2000 subscribers in 1946, while the society had grown to 452 members who were organized in several specialized committees [2].

In November of 1947, the Council decided to change the name of the Society to the “American Psychosomatic Society” [7]. It gave up the intention to “attempt to organize and direct or subsidize research in psychosomatic medicine or in any of the allied disciplines. It was felt that in the present phase of the development of psychosomatic medicine the Society's function could be best expressed by offering itself as a forum in which the various groups working in psychosomatic medicine could present the results of their investigations and in which they would be informed of work being done in their respective fields.” [8].

## Decades of development

By 1951 the membership had risen to >500 [9].

However, in the late 1950s the APS and North American psychosomatic medicine were perceived as being in a crisis. Zilboorg [5], although calling psychosomatic medicine the “most conspicuous and dramatic postwar development” stated that “whether psychosomatic medicine is here to stay as a specialty, or a subspecialty, is doubtful.” and “The future of this synthesis (of psychoanalysis, psychiatry, and medicine, CH-L) is as yet neither clear nor otherwise determined.”

In his Presidential Address, Wittkower [10] reported on an analysis of studies published in Psychosomatic Medicine over 20 years and found that the percentage of articles with sole or senior authors who were psychiatrists had increased from 60% in the first years to 74% in 1954–58 and that the percentage of neurologists or other medical specialists had in the same time dropped from 22% to only 7%, while the contributions of epidemiologists and of social scientists had remained “regrettably small”. Wittkower also observed a shift from more clinical to more experimental and conceptual research and stated that “there is a growing cleavage noticeable in psychosomatic publications between those with more and more psychiatry and less and less physiology, and those with more and more physiology and less and less psychiatry.” [10] In his conclusion, Wittkower assumed that as a result of “fragmentation into particularistic interests” and a “decline in interest in psychosomatic medicine on the part of physicians” psychosomatic medicine had “entered a crisis that threatens its very existence. It has been stated often that psychosomatic medicine is not a specialty, but an approach with a common focus on which workers in different fields converge, and that it works toward its own dissolution— assuming general acceptance of a multifaceted perspective of health and illness, with acknowledgment of the relevance of emotional factors to, and the pluricausality of, illness. The question may be raised whether this phase in the development of our field has been reached, and whether, consequently, psychosomatic medicine as such has lost its usefulness as a collaborative endeavor.” ([10]; p.313) Wittkower tended to answer this question to the negative and referred to – at that time – recent integrative concepts of the field by Grinker, Bandler, Engel and others, calling for a comprehensive, multidisciplinary approach to the field.

Interestingly it was in the same year of perceived crisis in North American psychosomatic medicine that the Japanese Psychosomatic Society held its inaugural meeting in Tokyo and started its impressive development. In preparation for that meeting, the APS was requested to answer a number of questions about the nature of psychosomatic medicine and psychosomatic disease. In an Editorial, Binger [11] answered as follows: “Psychosomatic medicine is another name for comprehensive or holistic medicine. In other words, it is a point of view toward the study of illness and disease and an approach toward research. It includes the individual's reactions to his illness and its implications for his personal and social life as well as the effects of these upon the functioning of his body. … there is no such thing as psychosomatic disease. All disease can be looked at from this point of view. … The goal of psychosomatic research is to explore and clarify the interrelationship of psychological processes and physiological and pathological ones, with the hope of achieving a clearer understanding of what illness consists of. … The proper future of psychosomatic medicine is its disappearance, with replacement by a true holistic or comprehensive medicine.”

However, little progress seems to have been made towards this goal in the subsequent ten years. In his Presidential Address ten years later Mason [12] regretted that “realistically, we must face the fact that the psychosomatic approach has not as yet had the sweeping, revolutionary impact on medicine of which it appears capable.” He proposed strategies for improved multidisciplinary research on the mechanisms involved in psychosomatic illnesses to cover the complexity of processes involved. Such multidisciplinary research might require collaborations on a national rather than local level. He concluded that “The future almost certainly will require some revolutionary departures from conventional practices in order for the integrative approach to become firmly established in medicine. If this is to be done in the near future, almost certainly we here in this room and our successors must do it.” ([12]; p. 439) .

Interestingly, Presidential Addresses from subsequent years (eg, [13–16]) were more likely to report on specific research methods, original study data and advancements in psychosomatic medicine rather than discussing the future of psychosomatic medicine or research per se. However, in 1979 Graham [17] discussed “Psychosomatic medicine’s place in medicine”. He agreed with the former APS President George L. Engel’s seminal *Science* paper [18], where Engel stated that “Psychosomatic medicine-the term itself a vestige of dualism-became the medium whereby the gap between the two parallel but independent ideologies of medicine, the biological and the psychosocial, was to be bridged. Its progress has been slow and halting, not only because of the extreme complexities intrinsic to the field itself, but also because of unremitting pressures, from within as well as from without, to conform to scientific methodologies basically mechanistic and reductionistic in conception and inappropriate for many of the problems under study. Nonetheless, by now a sizable body of knowledge, based on clinical and experimental studies of man and animals has accumulated. Most, however, remains unknown to the general medical public and to the biomedical community and is largely ignored in the education of physicians. … The fact is that medical schools have constituted unreceptive if not hostile environments for those interested in psychosomatic research and teaching, and medical journals have all too often followed a double standard in accepting papers dealing with psychosomatic relationships” ([18]; p. 134). Graham therefore recommended the Society to “return a little towards its former emphasis on the understanding of 'medical' disease. This suggestion is no way intended to disparage basic research.” [17] Based on that better understanding Graham called for “more evidence that psychological approaches are of therapeutic value in patients with medical diseases” with “particular emphasis on improvements in the technique of therapeutic interviewing” [17].

As time progressed, Jenkins [19] found psychosomatic medicine in a process of differentiation – with an increasing number of subspecialty journals addressing the field which had previously only be covered by the APS journal *Psychosomatic Medicine* and that “many more general scientific and medical journals regularly accept papers which are basically psychosomatic than was the case in earlier years.” The APS membership had grown to 950 by 1984. However, Jenkins stated that psychosomatic research still paid insufficient attention to individuals’ environments, cultural factors and to persons in childhood, old age or minority populations, as well as to treatments and rehabilitation for major illnesses, which he considered desirable for the future.

A membership survey conducted in 1987 [20] found that still >75% of APS members were MDs and the majority of them were psychiatrists. Only 13% of members were internists or pediatricians, while psychologists and sociologists made up 22.5%. However, the numbers only partially reflect the profound changes in membership that had occurred since the 1960s and continued to occur in later years: The initially strong influence of scientifically active internists slowly mellowed out as many internists retired or shifted their focus from research to teaching psychosomatic understanding and communication skills. Consultation programs in psychiatry were developed and many consultation psychiatrists turned away from the APS to join the Academy for Psychosomatic Medicine (APM), founded in 1954. The APM as a more clinically oriented society provides consultation-liaison psychiatrists with research findings directly helping them with clinical decision making, while the APS increasingly focused on research in psychobiological mechanisms. This split partly reflected changes in funding for medical schools in the U.S., which left little time for physicians to engage in research. Physicians who wanted to do research needed to obtain grant funding. Yet this was difficult to obtain without sufficient training in research methodologies, which is not routinely provided during medical education. In psychology new areas of research, namely health psychology and behavioral medicine with their organizations, competed with APS for clinically oriented members and impact. Taken together these changes led to a gradual reduction in clinical integration and intervention research.

APS’ 50^th^ anniversary was celebrated with a large international meeting held in New York in 1992. At that time, the Society had, in 1986, appointed a professional manager and was financially healthy due to a new publisher and contract for the Journal *Psychosomatic Medicine* which generated substantially increased and reliable royalties. These changes gave the Society the opportunity to engage more in committee work and press relations. Awards and scholarship programs were created and the Society started a newsletter as one of several measures to keep members more actively involved [2]. The anniversary meeting was co-sponsored by 16 US and international societies of related disciplines. Some 40% of attendees came from outside North America and approximately 50% were MDs. According to the conference organizers, the program reflected “the transition from the study of a disease which had been labeled ‘psychosomatic’ to studies of psychobiologic processes.” [2] However, a major focus of the meeting was also on consultation-liaison psychiatry and a two-day preconference was dedicated to hypnosis.

However, despite several efforts to keep the balance between psychosomatically-oriented internists or other medical specialists on one hand and consultation-liaison psychiatrists on the other, and between clinicians and more basic researchers, the clinical focus in APS diminished further in the following two-and-a-half decades. The position of Ph.D.s in the Society was strengthened in order to meet the interests of health psychologists and basic researchers. This undoubtedly led to a strong research record of the APS membership whose publications now appear in highly ranked medical and psychological journals, in addition to the continuously high quality of research published in *Psychosomatic Medicine*. One important initiative in this context focused on advancing neuroscience in psychosomatic research by organizing dedicated symposia at APS meetings, reviewing relevant literature in several articles in Psychosomatic Medicine and encouraging original research by, eg, the donation of a dedicated annual award [21].

M.D. members lost their majority in the Society in 2001 and never again reached a portion of >50% of APS members. Since 2009 M.D.s make up some relatively stable 30% of the membership. The decline in the numbers of (often older) M.D. members went along with an increase in younger members (<47 y/o) from 25% in 2000 to a maximum of 46% in 2014 and a related increase in members holding neither an M.D. nor Ph.D. degree from 10% in 2000 to approximately 20% in recent years (unpublished data). Over the last 20 years, APS Presidents have been alternating at approximately equal rates between M.D.s and Ph.D.s [6].Young members and international researchers have been attracted successfully by a variety of travel scholarships and similar programs while all efforts of increasing the number of M.D. members again showed little effect.

In contrast to this development in APS, institutionalized psychosomatic medicine gained increased credit by the creation of a subspecialty in “Psychosomatic Medicine” for psychiatrists in 2005, mainly reflecting consultation-liaison psychiatry. However, the new specialty found little resonance in the APS and most clinical psychiatrists specialized in “Psychosomatic Medicine” are now organized in the Academy of Psychosomatic Medicine, while the majority of APS members is nowadays made up by non-physician researches.

## The quest for APS’ identity in the 21^st^ century

The decreasing number of M.D. members and the parallel increase in basic “mechanistic” research (a term that still had negative connotations in earlier years of the society; see [18]) almost inevitably led to long-lasting discussions about a possible change of the society’s and its journal’s names.

As early as in 1960, Wittkower [10] had stated in his Presidential Address mentioned above that “The future of psychosomatic medicine, to repeat, lies in a multidisciplinary approach. However, it may be seriously questioned whether the outcome of such a comprehensive multidisciplinary study would still be justifiably and adequately covered by the term 'psychosomatic'.” [10].

In 1977, an ad-hoc committee was created to consider a name change or a subtitle for the journal “*Psychosomatic Medicine*”. After several months of discussion the committee decided to keep the name of the journal and also not to add anything to it. However, with its 70^th^ anniversary in 2009, the editors of *Psychosomatic Medicine* – with the approval by the APS Council – finally added the subtitle “*Journal of Biobehavioral Medicine*” to the (unchanged) name of the journal. When the subtitle was added to the journal, the editors stated that it aimed at helping the journal to reach out to the broader medical community and to reconcile “the journal’s identity with its 21st century content.” [22]. However, the new subtitle hasn’t led to a measurable increase in the journal’s impact factor over the subsequent years. The editors continued that “Some day in the not-too-distant future, this journal may no longer be called Psychosomatic Medicine. It may eventually become Biobehavioral Medicine, or perhaps The Journal of Biobehavioral Medicine” [22].

This occurred during a phase of discussions within the membership of APS whether it should not better change its name in total. Several members had several reasons for considering a name change. Its partly negative connotation among somatic clinicians and the lay public, who often use it in a stigmatizing way, was one key argument. Unlike other countries such as Germany or Japan, the term “psychosomatic” has negative connotations among the lay public and many health practitioners in the United States, where it is often equated with imaginary symptoms rather than (measurable) mind-body interactions. In addition, the term was thought to perpetuate the mind-body dualism it originally intended to overcome by, eg, ignoring the social dimension. Finally, many behaviorally or biologically oriented researchers perceived “psychosomatic” as too closely linked to “psychoanalytic”. In fact, psychoanalytic psychiatry while having been at the heart of APS in its early years has been the theoretical and clinical background of only a small minority of members in recent years. A majority of members now understand themselves as behavioral, biological or “biobehavioral” researchers, as epidemiologists, neuroscientists or physician scientists. The name change discussions started in the late 1990s and several alternative names were brought up during the process. However, none of the alternative names developed sufficient traction to justify the abandonment of history and the costs associated with rebranding.

The discussion only came to an end after a membership vote in 2013, where members were finally invited to decide whether they wanted to keep the historical name or rename the society to “Association for Biopsychosocial Medicine”. Although “biopsychosocial” was widely acknowledged to cover the interpersonal and social aspects of psychosomatic medicine better than the original term, a majority finally voted in favor of keeping the original name [23]. This does not necessarily indicate broad support for the term “psychosomatic”. However, the predominating opinion appeared to be that it might be more favorable to educate the public about the real focus of the field and the research APS members are conducting, so that the term “psychosomatic” might regain the positive holistic implication it originally had, rather than adopting one of the alternative proposals which also all appeared suboptimal to many members and might have raised new problems.

## APS’ current mission, goals and strategies

So APS is still the American Psychosomatic Society when it comes to celebrating its 75^th^ anniversary in 2017/18. However, APS has, in early 2016, refined its goals, adapted a new mission and strategic goals and launched a number of activities to achieve those goals [24, 25].

The mission statement as published on the APS website (www.psychosomatic.org) now reads: *“To advance and integrate the scientific study of biological, psychological, behavioral and social factors in health and disease.”*

Based on the mission and APS’ core values, three strategic goals were prioritized:

- ***Scientific excellence*** *:* Advance scientific excellence in psychosomatic medicine
- ***Clinical relevance*** *:* Increase clinical and public health relevance of biopsychosocial research
- ***Vibrant and diverse membership*** *:* Ensure an engaged membership balanced by disciplines, career stages, national/ethnic backgrounds, clinical vs. basic researchers.

Several ongoing and newly adopted strategies are underway to reach these goals and each of the strategies is supported by one or more specific tactics.

Strategies related to the goal of scientific excellence include:

- Publish papers in *Psychosomatic Medicine* that would be routinely cited in Introduction and Methods sections of papers
- Provide training to students and fellows on how to review for the journal
- Integrate affect science and psychobiology to advance scientific rigor and clinical relevance in psychosomatic medicine. This strategy is expected to build on the initial intention of the society to study associations between “Emotions and Bodily Changes” [1] and redefine it for the 21^st^ century. It is also intended to increase clinical relevance of research. Tactics include, among others
  - A special topic meeting in the fall of 2017, focusing on “Emotions in Social Relationships – Implications for Health and Disease”, together with a special issue in Psychosomatic Medicine and an Anniversary Award related to the topic of the meeting
  - Invited Symposia and Speakers at Annual Meetings
  - Experienced Investigator Colloquium at Annual Meeting
- Setup infrastructure for collaborative research: APS will join and support an initiative for creating a Behavioral Medicine Research Council (BMRC), aiming at integrating efforts across organizations to clarify major biobehavioral research questions and guiding scientific directions to advance and coordinate clinically relevant science in biobehavioral medicine.
- Collaborate with the Consortium of Social Science Associations (COSSA [26]), providing a united voice for social and behavioral science in US political affairs.

The following strategies are related to the goal of clinical relevance:

- Education: APS is working with authors of educational videos and other materials for the APS website to translate research findings into clinical application. APS members are also working on a comprehensive educational textbook on psychosomatic medicine and on educational programming during Annuals Meetings
- APS is liaising with the Integrated Primary Care Alliance(IPCA) to improve biopsychosocial understanding and practice in primary care settings
- A dedicated Clinical Series is being prepared for *Psychosomatic Medicine*
- APS started cross-advertizing for events with the Academy of Psychosomatic Medicine as a mainly clinically-oriented partner society.

As related to the goal of fostering a vibrant and diverse membership the following initiatives are underway:

- Several ongoing scholar and travel awards mainly targeting trainees and junior scientists from the US and worldwide
- A new international lab-to-lab exchange program [27] for which APS is collaborating with the International Society for Behavioral Medicine (ISBM) and the Society for Health Psychology
- The anniversary awards mentioned above, which offer an opportunity for senior and mid-career researcher to get their outstanding work recognized beyond the existing APS awards such as the Herbert Weiner Early Career Award, the MacLean Award for outstanding neuroscience research in Psychosomatic Medicine, the Patricia A. Barchas Award for Sociophysiology, the Distinguished Scientist Award and the Donald Oken Fellowship honoring a distinguished consultation-liaison psychiatrist or internist who hasn’t previously attended an APS meeting.

## Conclusion

This year, APS is celebrating its 75^th^ anniversary [28], starting with the 2017 Annual Meeting which was held in Sevilla, Spain on March 15–18, 2017, following a tradition adopted in 2002 to hold an Annual Meeting outside North America every 5 years to facilitate interaction with international researchers and clinicians in the field., Anniversary celebrations are continuing through the special topic meeting on “Emotions in Social Relationships – Implications for Health and Disease”, which will take place in Berkeley, California, on October 20-21^st^, 2017 and culminate in a series of events during the 2018 Annual Meeting, which will take place in Louisville, Kentucky on March 7–10, 2018. For the anniversary celebrations, APS is currently conducting some research into its history, taking advantage of a rich pool of archived materials ranging back to the very beginnings of the society, collecting historical memories by interviews with senior members and addressing historical aspects during the jubilee meetings. Readers are cordially invited to submit historical materials to the APS national office at info@psychosomatic.org and to join us in celebrating the 75^th^ anniversary and developing American and international psychosomatic medicine into the future.
